# A Review on Polyphenols in *Salicornia ramosissima* with Special Emphasis on Their Beneficial Effects on Brain Ischemia

**DOI:** 10.3390/nu15030793

**Published:** 2023-02-03

**Authors:** Ana M. Nájar, Marina Romero-Bernal, Carmen del Río, Joan Montaner

**Affiliations:** 1 Institute of Biomedicine of Seville (IBiS), Hospital Universitario Virgen del Rocío, CSIC, Universidad de Sevilla, 41013 Seville, Spain; 2 Department of Neurology, Hospital Universitario Virgen Macarena, 41009 Seville, Spain

**Keywords:** *Salicornia*, stroke, brain ischemia, polyphenol, neuroprotection

## Abstract

There has been an increasing interest in the consumption of halophytes as a healthy food in the last few years. *Salicornia ramosissima* is a seasonal Mediterranean halophyte with an interesting profile of bioactive compounds, including more than 60 identified polyphenols with a broad range of biological activities. Accumulating evidence supports the role of dietary polyphenols in the prevention of cardiovascular diseases, such as stroke. Stroke is the second cause of death worldwide and it is estimated that a substantial proportion of stroke incidence and recurrence may be prevented by healthier dietary patterns. Here, we have grouped the phenolic acids and flavonoids identified in *S. ramosissima* and reviewed their potential protective effect on brain ischemia, which are mostly related to the reduction of oxidative stress and inflammation, the inhibition of cell death pathways and their role in the preservation of the vascular function. Despite the fact that most of these compounds have been reported to be neuroprotective through multiple mechanisms, human studies are still scarce. Given the safe profile of polyphenols identified in *S. ramosissima,* this halophyte plant could be considered as a source of bioactive compounds for the nutraceutical industry.

## 1. Introduction

Halophytes comprise more than 350 annual and perennial species capable of tolerating the severe environmental condition of high salinity. In recent years, these plants have aroused interest as a valuable environmental resource for their ability to generate biomass in drought and salinity and a high resistance to conventional plant diseases. Moreover, halophytes contribute to carbon stabilization and play a role in soil phyto-desalinization and phytoremediation [1]. In addition to these benefits, halophytes have been attributed with health-promoting effects, including anti-inflammatory and antioxidant activities [2]. 

*Salicornia ramosissima*, also known as glasswort, is an annual halophyte of the *S. europaea* agg that can be found on the coastline of Europe, including Portugal and Spain, where its aerial parts are frequently consumed as a fresh vegetable [3]. More recently, the use of halophytes has been suggested as an alternative natural solution to reduce the sodium content of food products that are traditionally produced using salt [4]. *S. ramosissima* has a good nutritional profile, being a source of mineral and fiber [5], but also bioactive compounds. synthesized in response to abiotic stress due to high salinity and UV radiation, such as polyphenols, dietary consumption of which is associated with many health benefits.

Improving nutritional lifestyle is a major strategy in controlling modifiable risk factors related to cardiovascular and cerebrovascular diseases. Stroke, which is the most common neurovascular disease, represents the second cause of death and the third cause of disability worldwide according to the World Health Organization. Hemorrhagic stroke is caused by bleeding blood vessels and represents about 15% of all stroke cases. Ischemic stroke, which is the most common type, is caused by the occlusion of a blood vessel by a thrombus, resulting in the local lack of oxygen and nutrients leading to brain cell death at the infarcted area [6]. Given the high demand for oxygen and glucose of the tissue, the local disruption of blood flow to a brain area leads to cell death by necrosis within minutes (ischemic core). This event is followed by the disruption of normal cell function in the surrounding area (penumbra), triggered by energetic failure, inflammation, acidosis, excitotoxicity, release of Reactive Oxygen Species (ROS) and the impairment of the blood–brain barrier (BBB), among other mechanisms [7].

Reperfusion therapies using thrombolytics or mechanical thrombectomy are the current available treatments for ischemic stroke [8]. Although reperfusion is certainly the therapeutic objective, it can also induce additional injury by triggering inflammation and oxidative stress [9]. Two major categories of experimental models of brain ischemia are used in the search for new therapies for stroke in a variety of animal species including rats and mice, namely global and focal ischemia. The most common method used for focal ischemia is middle cerebral artery occlusion (MCAO), which can be permanent or transient, in order to allow reperfusion. In global ischemia, multiple cervical vessels are temporarily occluded [10].

It has been shown that a substantial proportion of strokes can be attributed to unhealthy lifestyle behaviors [11] and up to 80% of stroke recurrence might be prevented by the application of a multifactorial approach that includes dietary modification [12]. Recently, our group reported that diet supplementation with *S. ramosissima* ethanolic extract with high content of polyphenols protected both flies and mice from the deleterious effects of ischemia [13]. Here, we aim to review bioactive compounds identified in the edible plant *S. ramosissima* and discuss their potential as therapeutic nutritional interventions for stroke therapy using the available bibliography on experimental models of brain ischemia. 

## 2. Natural Bioactive Compounds Found in *S. ramosissima*

Saline stress response in Salicornia species comprises major events in plant tissues including osmotic, anatomical and physiological adaptations, as well as metabolic changes that involve the production of secondary metabolites [14]. On the basis of their structure and chemical nature, there are three main groups of secondary metabolites biosynthesized by plants: (i) terpenes, (ii) phenolics and (iii) sulfur and nitrogen-containing compounds (glucosinolates and alkaloids, respectively) [15]. 

Phenolics are a broad group of secondary metabolites that range from an aromatic ring (bearing one or more hydroxyl substituents) to more complex examples. Plant phenolics are biosynthesized through the shikimic acid and phenylpropanoid metabolism pathways [16,17]. The biosynthesis of phenolic compounds in plants begins with the conversion of glucose to glucose-6-phosphate to produce either phosphoenolpyruvate (PEP) by glycolysis or erythrose-4-phosphate by the pentose phosphate pathway (PPP). PEP and erythrose-4-phosphate are used together to produce phenylalanine and tyrosine by the shikimic acid pathway, which involves seven sequential enzymatic steps. Phenylalanine and tyrosine are then channeled into the phenylpropanoid pathway to generate several phenolic compounds by means of five rate-limiting enzymes [15,17,18].

Among the compounds of interest in Salicornia species are polyphenols, characterized by the presence of phenol rings in their structure, ranging from simple molecules to complex polymers. Polyphenol subgroups include phenolic acids, flavonoids, lignans and stilbenes [14,17].

The main polyphenols found in *S. ramosissima* are phenolic acids and flavonoids, as shown in Table 1. However, it has been reported that the polyphenol content varies in response to salinity levels and other stress factors [14,19]. These compounds have been of interest in recent decades due to their therapeutic effect in different conditions, including a protective role in ischemia, anti-thrombotic effects and other health-promoting neurovascular benefits. 

## 3. Bioactive Compounds with Neuroprotective Effect against Brain Ischemia

### 3.1. Polyphenols 

#### 3.1.1. Phenolic Acids

Phenolic acids are formed by the substitution for hydrogen atoms on benzene rings by a carboxylic acid group and at least one hydroxyl [26]. Phenolic acids are the most commonly studied and a diverse class of plant polyphenols. High concentrations are found in a variety of plant-based foods such as seeds, skins of fruits and leaves of vegetables [27]. Phenolic acids are mainly divided into two sub-groups: hydroxybenzoic (HBA) and hydroxycinnamic acid (HCA), which are based on one-carbon side-chain (C6–C1) and three-carbon side-chain (C6–C3) structure, respectively. HBAs are found abundantly in oilseeds, cereals, coffee, cowpea, black currant, raspberry, squash shells and seeds, and blackberry. HCAs are sourced mainly from coffee, cherries, cereals, peaches, spinach, citrus juices and fruits, plums, tomatoes, potatoes and almonds [28]. Phenolic acids are abundantly consumed in regular human diet, with a daily intake of about 200–400 mg/day depending on the dietary regime [29,30], since these compounds are not evenly distributed in foods [31].

Gallic acid (GA, 3, 4, 5-trihydroxybenzoic acid) is among the best studied polyphenols and its antioxidant and neuroprotective properties have been widely investigated in vivo and in vitro [32]. Sun et al. revealed that GA pretreatment protected SH-SY5Y neuroblastoma cells against hypoxia/reoxygenation injury induced by sodium hydrosulfite (Na_2_S_2_O_4_) in a concentration-dependent manner. This in vitro experiment showed that pretreatment with GA reversed ROS-induced lipid peroxidation, decreasing malondialdehyde (MDA) levels, and reduced mitochondrial dysfunction by inhibiting the mitochondrial permeability transition pore (MPTP) opening. The transient MCAO (tMCAO) rat model also shows the neuroprotective effects of GA in vivo. GA intravenous treatment 20 min before the onset of ischemia decreased infarct volume and neurological deficits. GA reduced the release of cytochrome complex (Cyt C, an inducer of caspase-dependent death) to cytoplasm in the infarct area and apoptosis [33]. GA has also been reported to improve the BBB disruption caused by global ischemia/reperfusion (I/R), probably thanks to its antioxidant properties. In this study, it was demonstrated that treatment with GA once daily for 10 days before ischemia ameliorated depression and anxiety-like behaviors. GA also improved hippocampal electrical activity induced by I/R, which has long been hypothesized to play a role in cognition [34]. In addition, anti-inflammatory properties have been attributed to GA. Intraperitoneal GA treatment at different time points after tMCAO induction downregulated the expression of brain ionized calcium binding adaptor molecule-1 (Iba-1), which is a marker for activated microglia, the main immune effector cells in the central nervous system (CNS). Consistently, GA modulated cytokine production towards an M2 anti-inflammatory profile [35]. Interestingly, both studies found that microglial phenotype modulation plays a role in the GA neuroprotective effect on BBB [34,35]. 

As a powerful antioxidant, caffeic acid also exerts anti-inflammatory effects. Zhou et al. showed that treatment with caffeic acid for 5 days starting immediately before cerebral I/R ameliorated neurological symptoms, neuron loss in the ischemic core and the infarct volume 24 h after reperfusion. Moreover, during the chronic phase after reperfusion, caffeic acid also attenuated delayed injuries, especially the astrocyte proliferation in the boundary zone adjacent to the ischemic core. However, treatment with caffeic acid did not reduce the brain edema in the acute phase [36]. Similarly, Liang et al. revealed in a global cerebral I/R injury model in rats that pretreatment with a single dose of caffeic acid 30 min prior to ischemia can significantly improve learning and memory function as well as preserve neurons in the hippocampus in a dose dependent manner during the chronic phase after I/R. Authors showed that the mechanism of protection of this phenolic acid is related to an increase of superoxide dismutase (SOD) activity, inhibition of Nuclear Factor Kappa ꞵ (NF-κꞵ) activation in the nucleus and reduced MDA content [37]. Interestingly, both studies found that 5-lipoxygenase (5-LOX, a pro-inflammatory mediator) was inhibited by caffeic acid. Because inflammation and oxidative stress are involved in neuronal injury, the inhibition of 5-LOX by caffeic acid may partially explain its therapeutic effect on brain ischemia. 

Chlorogenic acid (CGA) is the ester of caffeic acid and quinic acid and has been reported to have antioxidant [38], anti-inflammatory [39] and anti-apoptotic properties [40]. In this sense, intraperitoneal administration of CGA to rats 2 h after MCAO reduced brain infarction and attenuated neurological dysfunction and brain edema. CGA treatment was shown to impede caspase activation, which is a crucial mediator of apoptosis, but also alleviated the increase in poly ADP-ribose polymerase (PARP) activity, a DNA repair enzyme involved in tissue damage during ischemia [41]. In a second study, these authors reported the role of CGA in the disruption of the neuroinflammatory cascade during I/R by reducing the activation of NF-κB, as well as glial cells’ reactivity, two main regulators of the production of proinflammatory cytokines [39]. CGA is quickly absorbed in the rat stomach in its intact form [42] and it has been estimated that, in humans, one third of CGA is absorbed in the small intestine [43]. Accordingly, oral administration of CGA had a dose-dependent neuroprotective effect on global brain I/R in rats, which was abolished in the presence of an inhibitor of the nuclear erythroid 2-related factor 2 (Nrf2) pathway. The oxidative stress reduction improved the brain tissue pathology and decreased cortical cell apoptosis. Moreover, CGA mitigated memory impairment and reversed the inhibition of the neurotrophic factors brain-derived neurotrophic factor (BDNF) and nerve growth factor (NGF), alleviating nerve injury [44]. In agreement with this, Zheng et al. recently reported the protective role of CGA pretreatment on rat neonatal stroke by regulating the Nrf2-NF-κB signaling pathway [45].

Ferulic acid (FA, 4-hydroxy-3-methoxycinnamic acid), a derivative of cinnamic acid, is one of the main active ingredients used in traditional Chinese medicine. Pharmacological studies have shown this phenolic acid to have a variety of biological activities, especially on oxidative stress and inflammation [46], endothelial injury [47] and regulation of lipid metabolism [48]. FA was reported to exert neuroprotective effects against experimental stroke by activating p38 mitogen-activated protein kinase (MAPK) signaling, which is involved in cell growth and differentiation, among other cell processes. Intravenous administration of FA using different treatment regimes (pre- and post-ischemia) reduced the area of cerebral infarction and suppressed inflammation and apoptosis after tMCAO in rats [49]. The anti-apoptotic effect of FA in brain ischemia was also confirmed by Ren et al., who reported that FA treatment for 5 consecutive days after ischemia in a global brain I/R model in rats improved spatial learning and memory deficits, increased the generation of the cellular antioxidant defenses (SOD and GSH) and reduced MDA levels. They suggested that FA exerts anti-apoptotic effects by increasing B-cell lymphoma-2 (Bcl-2) levels and inhibiting caspase-3 and Bax-mediated apoptotic signaling in the hippocampus in a concentration-dependent manner [50]. Bcl-2 is a key regulator of the mitochondrial apoptosis pathway localized in the outer membrane of mitochondria and forms a heterodimer with the apoptotic protein Bax. This prevents Bax homodimerization and triggers the activation of terminal caspases, releasing Cyt C and ROS into the cytoplasm and promoting apoptosis [51].

Another cinnamic acid derivative, *p*-coumaric acid (PC, 4-hydroxycinnamic acid), has also been studied for the treatment of brain ischemia in vivo. Guven et al. tested the effect of intraperitoneal PC 5 min after focal brain ischemia in rats and revealed an improvement in neurological deficit, decreased MDA levels and increased SOD activity, confirming the alleviation of oxidative stress levels by PC treatment after brain I/R injury. This study suggested that PC activates both extracellular signal-regulated kinase (ERK) and Akt signaling pathways, leading to the inhibition of apoptosis [52]. Regarding its oral availability, PC is rapidly absorbed by the monocarboxylic acid transporter present in colon epithelial cells in an intact form [53]. A second study confirmed the neuroprotective effect of PC when administered orally prior to the induction of global brain I/R in mice. PC treatment for two weeks reduced infarction volume and improved brain oxidative status, revealed by a decrease in MDA and increased levels of the antioxidant enzymes SOD and catalase [54].

Vanillic acid (VA, 4-Hydroxy-3-methoxybenzoic acid) is a dihydroxybenzoic acid derivative used for treating various ailments. VA has shown neuroprotective effects in in vivo models of ischemia. VA treatment for 14 days prior to focal brain I/R in rats reduced both neurological deficit and cerebral infarct volume. VA also downregulated the expression of NF-κB-related downstream inflammatory genes and showed excellent free radical scavenging activity [55]. Furthermore, in a similar study using global ischemia, VA decreased cognitive impairments, probably as a consequence of reduced inflammation and apoptotic cell death in the CA1 region of the hippocampus [56]. 

The neuroprotective effect of syringic acid (SA, 4-hydroxy-3,5-di-methoxybenzoic acid), a polyphenolic derivative of benzoic acid, has been demonstrated using in vitro and in vivo studies. SA pretreatment exerted antioxidant effects in a model of hippocampal neurons subjected to oxygen-glucose deprivation/reoxygenation (OGD/R)-induced cell injury, possibly through the JNK and p38 signaling pathways. Furthermore, SA attenuated the loss of cell viability and apoptosis [57]. Additionally, SA treatment showed similar effects when administered 5 min after permanent MCAO (pMCAO), ameliorating oxidative stress and reducing neuronal degeneration [58]. 

Other less studied phenolic acids in the stroke field include sinapic acid (SP, sinapine) and ellagic acid (EA). SP is a small naturally occurring hydroxycinnamic acid and its effects have been studied in global brain I/R. Intraperitoneal treatment with SP protected rats against neuronal damage, especially preserving hippocampal CA1 neuronal density, which is critical in learning and memory processes and consistent with an improvement in cognitive decline [59]. On the other hand, EA is a low-molecular-weight polyphenol derived from fruits, vegetables and nuts. EA exhibited neuroprotective effects through the Bcl-2 pathway both in vitro and in a global brain I/R injury in rats. EA treatment prevented primary neuron death and decreased the ratio of Bcl-2/Bax expression, reducing apoptosis. In vivo data showed that EA treatment significantly reduced infarct volume using a prothrombotic stroke model. EA was shown to increase the number of Bcl-2 positive neurons and the Bcl-2/Bax heterodimer ratio in the ischemic semi-dark zone and ameliorated the neurological deficit [60]. 

Interestingly, we recently found cannabidiolic acid (CBDA) to be present in *S. ramosissima* samples [13]. CBDA is the carboxylated precursor of CBD, a well-known anti-inflammatory and neuroprotective cannabinoid that was shown to reduce brain damage and neuronal loss in adult and neonatal models of ischemic stroke [61,62]. However, CBDA has been less studied than its decarboxylated form and its effects on ischemia have not yet been studied.

The neuroprotective effect of some derivatives of phenolic acids identified in *S. ramosissima* have also been described. The administration of 4-hydroxy-3,5-di-tret-butyl cinnamic acid, a cinnamic acid derivative, contributed to the restoration of energy-producing mitochondrial function in the hippocampus as well as ameliorated oxidative stress and apoptosis in rats submitted to pMCAO [63]. 

Although not all *S. ramosissima* phenolic acids have been studied in experimental models of cerebral ischemia, numerous studies support the therapeutic activity of this type of compound in different diseases. This is due to the large range of biological effects they display, which include inhibition of intracellular Ca^2+^ concentration and the consequent mitochondrial damage and free radical production that ultimately leads to the initiation of neuronal death processes, as well as anti-inflammatory properties, through the inhibition of microglial activation and the modulation of pro-inflammatory mediators such as LOX-5. Table 2 summarizes the available in vivo studies analyzing the effect on brain ischemia of phenolic acids present in *S. ramosissima.*

#### 3.1.2. Flavonoids

Flavonoids, like phenolic acids, constitute one of the largest subgroups of polyphenols. High amounts of these compounds can be found in fruits and vegetables, such as citrus fruit, berries and apples, as well as seeds, legumes and beverages of plant origin [64]. Among flavonoids’ functions in the plant, they play a role in response to environmental factors, such as the coloration of fruits and flowers, and participate in the defense against UV radiation and pathogen infection. These compounds are found in plant vacuoles and their chemical structure consists of a skeleton along with three rings (C6-C3-C6), which are called A, B and C [65]. 

Regarding their classification, flavonoids are grouped according to their chemical structure, taking into account the degree of unsaturation and oxidation of the rings and the carbon of the C ring on which the B ring is attached. In this way, subgroups include flavanones, present mainly in citrus; flavanols, present in eucalyptus and buckwheat leaves; flavones, found in mint, chamomile and ginkgo biloba; chalcones, which lack the C ring and are found in tomatoes or strawberries; anthocyanins, responsible for the red and blue pigment of plants and therefore found to a greater extent in currants, grapes, strawberries or blueberries; and iso-flavonoids, mainly found in leguminous plants [66]. 

Many flavonoids described in *S. ramosissima* have been studied for their antioxidant activity. In addition, some have shown neuroprotective potential. Table 2 summarizes in vivo studies on the effect of flavonoids present in *S. ramosissima* on brain ischemia. This is the case for chrysin, a flavone which behaves as an antioxidant and anti-inflammatory compound, preventing neurotoxicity in vitro [67]. Moreover, oral administration of chrysin three weeks prior to brain ischemia in rats improved sensorimotor and memory parameters, as well as increased hippocampal cell survival in adult male Wistar rats [68]. The mechanisms by which this flavonoid exerts neuroprotection are varied. First, chrysin has been shown to maintain the integrity of the cell membrane, thereby lessening necrosis [67]. Oral administration prior to tMCAO in mice was also reported to decrease infarct volume and neurological deficit through reducing oxidative stress by dropping the formation of ROS, as well as behaving as a scavenger for O_2_. Moreover, anti-inflammatory activity has also been described, achieved by two routes; firstly, the inhibition of the expression of proinflammatory genes such as iNOS and COX-2 and, secondly, through the suppression of the NFκB pathway [69]. 

One of the most commonly studied flavonoids is quercetin, which is involved in several plant physiological processes, such as seed germination, photosynthesis and plant growth. Quercetin and its derivatives can present different biological activities due to modifications at significant positions of the quercetin molecule, such as glycosylation or methylation [70]. Quercetin was shown to protect human brain microvascular endothelial cells from I/R in vitro. At the cellular level, quercetin promoted cell viability and induced cell migration and angiogenesis. Quercetin induced the activation of the protective Keap1/Nrf2 signaling pathway, and consequently reduced ROS formation. Moreover, this flavonoid may have the ability to maintain the integrity of the BBB by sustaining tight-junction protein expression [71]. The protective effect on the neuro-vasculature has also been reported in vivo. In addition to reducing infarct volume and neurological function, intracerebroventricular injection of quercetin 30 min before global ischemia in rats was able to maintain the BBB integrity, including reduced permeability and ultrastructural alterations. Quercetin was found to activate the canonical Wnt/β-catenin signaling, which is involved in cell proliferation [72]. Unfortunately, oral bioavailability of quercetin is poor and can be affected by many factors such as glycosylation, solubility, vitamin C status or food matrix [73]. 

Within the flavonol subgroup, kaempferol, has been shown to protect against endothelial damage in vitro by lessening oxidative stress, specifically by reducing the levels of ROS and NO [74]. Kaempferol was also shown to protect against neurovascular disease in vivo. Preventive treatment by gavage for one week significantly reduced the volume of the infarct area as well as the neurological deficit score 24 h after tMCAO in rats. The mechanism of protection involved the regulation of various pathological pathways related to oxidative stress and inflammation, including Nrf2, Akt, NF-kβ and Gsk3β [75,76]. Similarly, two glycoside derivatives, namely kaempferol-3-O-rutinoside and kaempferol-3-O- glucoside, were found to be effective in the reduction of brain infarct by reducing brain cell inflammation mediated by the inhibition of NF-kβ and STAT3 [77].

Naringin, which is responsible for the bitterness of grapefruit and citrus skin, has also been reported to be neuroprotective in I/R models. It was shown in PC12 cells that naringin treatment decreased oxidative stress and apoptosis after OGD, partially attributed to increased Bcl-2 and decreased Bax levels. In the same way, naringin modulated several pathways involved in the inflammatory process, such as NF-kβ, Akt or mTOR [78]. Similarly, naringin promoted cell viability and reduced apoptosis after OGD in rat primary neurons, probably related to the activation of the Akt pathway [79]. Furthermore, prophylactic treatment with intraperitoneal naringin for seven days before tMCAO in male rats decreased the infarct volume in the brain, as well as apoptosis level [79]. Interestingly, naringin was found to cross the BBB when administered intravenously. Narigin can also exert neuroprotection when administered just at the beginning of reperfusion after tMCAO in rats and it was found to directly scavenge ONOO^−^ [80]. 

Naringenin is also a citrus flavonoid that can be found in beverages such as wine and coffee. Several studies have reported positive health effects, including a reduction in the vascular risk [81] or an anti-inflammatory activity [82]. Several in vivo studies have shown naringenin to offer neuroprotection in both ischemic and hemorrhagic stroke models. On the one hand, treating C57BL/6 male mice with naringenin by oral gavage 4 days before the induction of subarachnoid hemorrhage was shown to reduce the associated neurological deficit and cerebral edema. In addition, a reduction in inflammation and apoptosis was observed. Interestingly, the protective effect of naringenin in this model was abolished in the presence of either an AMPK or a Sirtuin-3 inhibitor [83]. On the other hand, oral administration of naringenin was shown to protect against experimental ischemic stroke. Preventive treatment for 21 days in male Wistar rats prior to tMCAO significantly reduced the infarct area and improved neurological deficit by suppressing NF-κB-mediated neuroinflammation [84].

Phloretin is a member of the dihydrochalcone flavonoids and is well known in dermatology for its anti-aging and depigmenting effects [85]. In addition, phloretin can exert a wide range of biological activities. Administration of intravenous phloretin was long ago proposed as a tool to drop the hazardous supply of blood glucose to the post-ischemic brain [86]. More recently, Liu and colleagues showed that intraperitoneal administration of phloretin for 14 days prevented neurological deficits and reduced infarct volume 24 h after tMCAO in rats. The mechanism underlying this protective effect seems to involve the activation of the Nrf2 defense pathway [87]. Accordingly, it has been reported that phloretin induced the activation of Nrf2 pathway mediated by the phosphorylation of AMPK, which consequently reduced the inflammatory phenotype of mice macrophages [88]. As happens with phloretin, phloridzin also plays a role in mediating glucose uptake in the brain [89] and has been shown to protect male mice from experimental stroke [90]. 

Epicatechin, a major polyphenol component of green tea and cocoa, has been shown to protect vasculature and to have neuroprotective capacity when studied in vivo. Epicatechin is rapidly absorbed in humans and has been reported to cross the BBB [91]. Oral consumption of pure (–) epicatechin enhanced flow-mediated vasodilation in humans [92] and several studies have demonstrated that oral administration of this flavonoid can prevent both ischemic [93,94] and hemorrhagic stroke [95] using rodent models. Consistently, all the studies have attributed this protective effect to the activation of the Nrf2 pathway.

As epicatechin, catechin is one of the major phenolic components of green tea. This flavonoid has been shown to have a great scavenging capacity, one of the mechanisms involved in its protective effect against ischemia [96]. One of the first studies with catechin on ischemia was carried out in 1998 in which Mongolian gerbil rodents were supplemented with catechin in drinking water for two weeks prior and one week after tMCAO. A dose-dependent increase in hippocampal cell viability was observed with the treatment. As expected, a decrease in O_2_^−^ was reported due to its scavenging activity, as well as a decrease in iNOS [97]. A different study later supported these results. After supplementing male Wistar rats with a green tea catechin extract in drinking water 5 days prior to tMCAO, authors reported a decrease in the infarct area of the brain and an improvement in the neurological deficit. The mechanisms underlying these effects include its scavenging activity as well as in the consequent effect on inflammation and vascular damage [98]. 

Rutin is a flavonoid glycoside with multiple pharmacological activities and substantial evidence has attributed its cytoprotective effect in ischemic tissues, including the brain. In vitro, treatment of PC12 cells prior to OGD attenuated the deleterious effect of hypoxia through a considerable decrease of ROS and the blockade of the apoptosis pathway by means of reduction of Bax and increase of Bcl-2 [99]. In addition, several studies have reported that oral supplementation with rutin can reduce the area of cerebral infarction and regulate the total amount of oxidative stress parameters, such as carbonylated proteins or antioxidant enzymes [100]. However, rutin has poor solubility, which can affect absorption and limit its oral application [101].

Apigenin, within the flavone subgroup, has beneficial effects on the vasculature which may reduce the risk of neurovascular disease. When given to rats, apigenin has behaved as a potent vasorelaxant [102]. Moreover, apigenin was shown to play a role in vascular inflammation [103] but also in platelet aggregation and secretion [104]. When used as a treatment against hypoxia in cellular models, apigenin exerted protection through the reduction of ROS and apoptosis [105] and induced angiogenesis by increasing the tube formation through a caveolin-1 dependent pathway [106]. Intraperitoneal treatment with apigenin attenuated brain damage after tMCAO in rats [105,107] and has been reported to preserve the proper functioning of the BBB by increasing caveolin-1 and to promote angiogenesis by rising VEGFS levels [107]. In a different study, intraperitoneal treatment with apigenin for 28 days following brain ischemia was shown to preserve nerve cells in rat brain hippocampus through multiple mechanisms involving histone deacetylases [108]. As happens with other flavonoids, apigenin is practically insoluble in highly polar solvents such as water and oral bioavailability is poor, as ingested apigenin is either excreted unabsorbed or rapidly metabolized after absorption [109].

Myricetin is mainly present in the glycoside form (*O*-glycosides) in food and beverages, such as tea and wine [110]. Among different biological actions, protective potential of myricetin against brain ischemia has been recently reviewed [111]. Using OGD in SH-SY5Y cells, Taheri et al. showed a beneficial effect of myricetin treatment on mitochondrial dysfunction and oxidative stress, two critical mechanisms of ischemic neuronal death [112]. In addition, myricetin was shown to reduce BV2 microglia proinflammatory phenotype [113] and decreased the enhancement of endothelial permeability and inflammation in human brain micro-vessel endothelial cell after OGD by regulating Akt and Nrf2 pathways [114], which could potentially protect the BBB breakdown after stroke. In experimental models of stroke in rats, oral administration of myricetin significantly reduced brain infarct volume and neurological symptomatology. It reduced apoptosis, inflammation and oxidative stress, and these protective effects were also related to Nrf2 and Akt pathways [115].

Taxifolin, also called dihydro-quercetin, is mostly found in milk thistle [116]. It is a flavanol-type flavonoid which has been shown to play a role in inflammation [117], cardiovascular risk [118] and neurological diseases, such as Alzheimer [119], among other health promoting benefits. Turovskaya et al. have shown that taxifolin can protect hippocampal neurons from OGD in vitro. Authors reported that taxifolin was able to reduce oxidative stress by lessening ROS production as well as enhancing cell viability by suppressing the apoptosis pathway through the increase in Bcl-2 [120]. These results are supported by in vivo studies using taxifolin. Intravenous treatment with taxifolin after pMCAO resulted in a dose-dependent decrease in the infarct area related to the reduction of COX-2 and iNOS, as well as a decrease in the adhesion protein ICAM-1. Moreover, a reduction in ROS was observed by the inhibition of NFκB [121]. 

Isorhamnetin is one of the main compounds of *Ginko biloba* leaves and *Hippophae rhamnoides* fruit [122]. Several studies support the use of this flavonoid as a therapeutic agent against cardio-cerebrovascular diseases [123,124]. At the cellular level, isorhamnetin treatment in HT22 mouse hippocampal neurons elicited a reduction of OGD-induced apoptosis, oxidative stress and inflammation. The mechanisms involving these effects are varied, including the increase in Akt, SIRT1, Nrf2, and HO-1 [125]. Intraperitoneal treatment with isorhamnetin attenuated brain damage after tMCAO in mice by reducing cerebral edema through the maintenance of tight-junction proteins at the BBB. In addition, isorhamnetin inhibited apoptosis through the down-regulation of caspase-3 and reduced oxidative stress and inflammation [126].

Tiliroside is a glycosidic flavonoid with several health promoting effects including anti-inflammatory [127] and antioxidant [128] activities. Regarding neuroinflammation, tiliroside was shown to inhibit deleterious NF-κB and p38 signaling pathways in LPS + IFNγ-activated BV2 microglia [129]. However, its role in cerebral ischemia has not yet been studied.

**Table 2:** Summary of in vivo studies on the neuroprotective effect of phenolic acids and flavonoids identified in *S. ramosissima* against cerebral ischemia.

Polyphenol	Model	Treatment	Observed Effects	Molecular Mechanism	Ref.
Gallic acid	Male SD rats	20 min before tMCAO (25, 50 mg/kg; i.v.)	Decreased infarct volume Anti-apoptosis Alleviated mitochondrial dysfunction	↓ Cyt C ↓ MPTP	[33]
	Male Wistar rats	Once daily for 10 days before transient 4VO (100 mg/kg; p.o.)	Ameliorated brain oxidative stress Improved the BBB disruption Alleviated anxiety, depression, locomotion behaviors	↑ SOD ↓ MDA	[34]
	Male C57BL/6J mice	30 min, 1, 12, 24, 48 and 72 h after ischemia in tMCAO (50, 100, 150 mg/kg; i.p.)	Reduced infarct area and edema Improved BBB disruption Anti-inflammatory Improved neurological function Inhibited microglial activation	↓ IL-1β, TNF-α, IL-6 ↑ IL-10 ↓ MMP-9 ↑ ZO-1, Claudin-5 ↓ Iba-1	[35]
Caffeic acid	Male SD rats	30 min before and from 0 h to 5th day after tMCAO (10, 50 mg/kg; i.p.)	Decreased infarct volume and neuron loss Ameliorated neurological dysfunction Attenuated late astrocyte proliferation	↓ 5-LOX	[36]
	Male SD rats	30 min before BCCAO combined with hypotension (10,30, 50 mg/kg; i.p.)	Preserved hippocampal neurons Anti-apoptosis Improved learning and memory function Reduced brain oxidative stress Anti-inflammatory	↑ SOD ↓ MDA ↓ 5-LOX ↓ NF-κBp65	[37]
Chlorogenic acid	Male SD rats	For 7 days before BCCAO (20, 100, 500 mg/kg; p.o.)	Reduced infarct volume and hippocampal neuron loss Anti-apoptosis Relieved nerve injury Ameliorated oxidative stress	↑ BDNF, NGF ↑ SOD, GSH ↓ MDA, ROS ↑ Nrf2/NQO-1/HO-1	[44]
	Male SD rats	2 h after pMCAO (30 mg/kg; i.p.)	Alleviated brain infarction and edema Anti-apoptosis Improved neurobehavioral deficits	↓ ROS, LPO ↓ Caspase-3, caspase-7 ↓ PARP	[41]
	Male SD rats	2 h after pMCAO (30 mg/kg; i.p.)	Ameliorated oxidative stress Inhibits the activation of astrocytes and microglia Anti-inflammatory	↓ ROS, LPO ↓ GFAP, Iba-1 ↓ NF-κB ↓ IL-1β, TNF-α	[39]
Ferulic acid	Male SD rats	Pre (2 and 4 h) and post (0,2 and 24 h) tMCAO (100 mg/kg; i.v.)	Alleviated brain infarction Anti-apoptosis Suppressed reactive astrocytosis Improved neurological deficits	↑ p38 MAPK/p90RSK/CREB/Bcl-2 signaling pathway ↓ GFAP ↓ Mitochondrial Bax ↓ Cyt C, Caspase-3	[49]
	Male SD rats	5 consecutive days after BCCAO (28, 56, 112 mg/kg)	Reduced hippocampal neuron loss Anti-apoptosis Improved memory deficits Anti-oxidative stress	↑ Bcl-2/Bax ratio ↓ Caspase-3 ↑ SOD, GSH ↓ MDA	[50]
*P*-coumaric acid	Male SD rats	5 min after pMCAO (100 mg/kg; i.p.)	Anti-oxidative stress Anti-apoptosis Ameliorated neurological deficits	↑ Nrf1, SOD ↓ MDA ↓ caspase-3, caspase-9 ↑ ERK, Akt ↓ ASK1	[52]
	Male ICR mice	2 weeks before BCCAO (100 mg/kg; p.o.)	Reduced infarction size Ameliorated brain oxidative stress Anti-apoptosis	↑ SOD, CAT ↓ MDA ↓ calcium	[54]
Vanillic acid	Male SD rats	Once daily for 14 days before tMCAO (50, 100 mg/kg; p.o.)	Ameliorated cerebral infarct volume Anti-inflammatory Ameliorated oxidative stress Reduce neurological deficits	↓ NF-κB ↓ IL-1β, IL-6, TNF-α ↓ MDA ↑ CAT, SOD	[55]
	Male Wistar rats	Once daily for 14 days before BCCAO (100 mg/kg; p.o.)	Reduced hippocampal neuron loss Anti-inflammatory Anti-apoptosis Reversed cognitive deficits	↑ IL-10, IL-6, TNF-α	[56]
Syringic acid	Male SD rats	5 min after pMCAO (10 mg/kg; i.p.)	Reduced histopathological changes Anti-oxidative stress Anti-apoptosis	↑ NRF1, SOD ↓ MDA ↓ Caspase-3, Caspase-9	[58]
Sinapic acid	Male Wistar rats	0 and 90 min aftertransient 4VO (10 mg/kg; i.p.)	Reduced hippocampal neuronal loss Improved cognitive impairment		[59]
Ellagic acid	Male SD rats	Once daily for 14 days before photothrombotic nerve injury (10, 30 mg/kg; p.o.)	Decreased the volume of infarction Decreased apoptosis Ameliorated neurological deficits	↑ Bcl-2	[60]
Chrysin	Male Wistar rats	Once daily 3 weeks prior to BCCAO, (10, 30, 100 mg/kg; p.o.)	Anti-apoptosis Attenuated memory impairment and sensorimotor parameters Ameliorated oxidative stress Decreased reactive hyperemia	↑ GPx ↓ MDA ↓ NO ↓ PGE2	[68]
	Male C57/BL6 mice	Once daily for 7 days before tMCAO (75 mg/kg; p.o.)	Reduced infarct volume and neuron loss Anti-inflammatory activity Anti-oxidative effects	↓ NF-κB, COX-2 ↓ iNOS ↑ SOD ↓ MDA ↓ GFAP, Iba-1	[69]
Kaempferol	Male SD rats	Once daily for 1 week before tMCAO (1.75, 3.49, 6.99 mM, 1 mL/kg; p.o.)	Decrease infarction volume Improved neurological deficit Anti-inflammatory Anti-oxidative effects	↑ Nrf2 ↑ Akt ↓ NF-kβ, Gsk3β	[75]
Naringin	Male SD rats	Once daily for 7 days before tMCAO (5 mg/kg; i.p.)	Decreased infarction volume Anti-apoptosis	↓ TNF-α ↓ IL-6	[79]
	Male SD rats	Once at reperfusion after tMCAO (80, 120, 160 mg/kg; i.v.)	Decreased infarction volume Reduced neurological damage Anti-apoptosis	↓ ONOO^−^	[80]
Phloretin	Male SD rats	Once daily for 14 days prior to tMCAO (20, 40, 80 mg/kg; i.p.)	Reduced infarct volume Anti-oxidative stress Reduced neurological damage	↑ Nrf2	[87]
Quercetin	Male SD rats	Twice daily for 3 days before BCCAO (25 μmol/kg; i.cv.)	Reduced hippocampal neuron loss Improved neurologic function Reduced brain edema Improved BBB permeability	↑ Claudin-5, ZO-1 ↓ MMP-9 ↑ Wnt/β-catenin signaling	[72]
Epicatechin	Male C57BL/6 mice	90 min prior to pMCAO (5, 10, 15 mg/kg; p.o.)	Reduced infarct volume and neuron loss Improved motor coordination Anti-oxidative stress	↑ Nrf2 ↓ Iba-1	[93]
	Male C57BL/6 mice	90 min prior to tMCAO (2.5, 5, 15, 30 mg/kg; p.o.)	Decreased infarction volume Improved neurological score	↑ Nrf2	[94]
Apigenin	Male SD rats	Once daily for 7/14 days after tMCAO (25 mg/kg; i.p.)	Reduced infarct volume Anti-apoptosis Improved BBB function Magnification in angiogenesis	↑ VEGFs ↑ Caveolin-1	[107]
	Male SD rats	Once daily for 7 days after tMCAO (25 mg/kg; i.p.)	Decreased infarction volume Improved neurological score	↓ ROS	[105]
	Male SD rats	Once daily for 25 days after tMCAO (20, 40 mg/kg; i.p.)	Decreased infarction volume Improved neuron viability Improve neurological score	↑ BDNF ↑ Syn-1	[106]
Myricetin	Male SD rats	Once daily for 7 days prior to pMCAO (1, 5, 25 mg/kg; p.o.)	Decreased infarction volume Anti-inflammatory Anti-apoptosis Decreased oxidative stress	↓ TNF-α, IL-6, IL-1β ↑ SOD ↓ MDA	[115]
Rutin	Male Wistar rats	Pretreatment for 21 days before tMCAO (25 mg/kg; orally)	Decreased oxidative stress Attenuated apoptosis Reduction in infarct size Improved neurobehavioral deficits	↑ GPx, GR, SOD, CAT, GSH ↓ H_2_O_2_, PC ↓ p53	[100]
Catechin	Mongolian gerbils	Once daily for 14 days prior and 7 days post tMCAO (5, 50 mg/kg; solved in drinking water)	Improved hippocampal neuron viability	↓ iNOS ↓O_2_^−^	[97]
	Male Wistar rats	5 days prior tMCAO (0.25%, 0.5%; solved in drinking water)	Decreased infarction volume Improve neurological score	↓ MDA ↓ iNOS ↓ NF-κB	[98]
Naringenin	Male Wistar rats	Once daily for 21 days prior tMCAO (10, 25, 50 mg/kg; p.o.)	Decreased infarction volume Improve neurological score Improved neuron viability	↑ SOD ↓ iNOS ↓ NF-κB,TNF-α	[84]
Phloridzin	Male ddY mice	0 and 6 h after tMCAO (40, 120, 200 mg/kg; i.p.) (10, 40 µg; i.c.v.)	Decreased infarction volume Improved neurological score Decreased FBG	↓SGLT	[90]
Taxifolin	Male Long-Evans rats	1 h after pMCAO (0.1, 1 µg/kg; i.v.)	Decreased infarction volume	↓ iNOS, COX-2 ↓ ICAM-1 ↓ NF-κB	[121]
Isorhamnetin	Male ICR mice	0 h after tMCAO (5 mg/kg; i.p.)	Decreased infarction volume Reduced brain edema Improved BBB function	↑ Claudin-5, ZO-1, occludin ↓ TNF-α, IL-6, IL-1β ↓ MDA	[126]

Symbols: (↑) increase; (↓) decrease. 4VO: 4-vessel occlusion; 5-LOX: 5-lipoxygenase; Akt: protein kinase B; ASK1: apoptosis signal-regulating kinase 1; Bax: Bcl-2-associated X protein; BBB: blood–brain barrier; BCCAO: bilateral common carotid arteries occlusion; Bcl-2: B-cell lymphoma 2; BDNF: brain-derived neurotrophic factor; CAT: catalase; COX-2: Cyclooxygenase 2; CREB: cAMP response element-binding; Cyt C: cytochrome complex; ERK: extracellular signal-regulated kinase; FBG: fasting blood glucose; GFAP: glial fibrillary acidic protein; GPx: glutathione peroxidase; GR: glutathione reductase; GSH: glutathione; Gsk3B: glycogen synthase kinase 3 beta; H_2_O_2_: hydrogen peroxide; HO-1: hemo oxigenasa-1; i.c.v.: intracerebroventricular injection; i.v.: intravenous injection; i.p.: intraperitoneal injection; Iba-1: brain ionized calcium binding adaptor molecule-1; IL: interleukin; iNOS: inducible nitric oxide synthase; LPO: lipid peroxidation; MAPK: mitogen-activated protein kinase; MDA: malondialdehyde; MMP-9: matrix metallopeptidase 9; MnSOD: manganese superoxide dismutase; MPTP: mitochondrial permeability transition pore; NF-Κꞵ: Nuclear Factor Kappa ꞵ; NGF: nerve growth factor; NQO-1: NAD(P)H quinone dehydrogenase 1; Nrf11: Nuclear respiratory factor 1; Nrf2: nuclear factor erythroid 2-related factor 2; O_2_^−^: superoxide anion; ONOO^−^: peroxy-nitrite; p.o.: per os; p90RSK: P90 ribosomal S6 kinase; PARP: poly ADP-ribose polymerase; PC: protein carbonyl; PGE2: prostaglandin E2; pMCAO: permanent middle cerebral artery occlusion; ROS: reactive oxygen species; SD: Sprague Dawley; SGLT: sodium-glucose transporter; SOD(2): superoxide dismutase (2); syn-1: synapsin 1; tMCAO: transient middle cerebral artery occlusion; TNF-α: tumor necrosis factor-α; VEGFs: vascular endothelial growth factors; ZO-1: zonula occludens-1.

## 4. Conclusions and Future Perspectives

In the present study, we have reviewed the effects of the different phenolic acids and flavonoids present in *S. ramosissima*. In vivo studies have reported that most of the identified polyphenols can ameliorate neuron loss in the ischemic core and reduce infarct volume. The associated functional improvements include the attenuation of neurological dysfunction and depression and anxiety-like behaviors, as well as balancing learning and memory processes and sensorimotor parameters. In addition, some of these polyphenolic compounds can mitigate BBB disruption and cerebral edema. Mechanisms underlying these health benefits include polyphenols’ powerful ability to eliminate ROS, but also their action on different signaling pathways involved in oxidative stress, inflammation and apoptosis.

In addition to the aforementioned properties, polyphenols have shown to exert neuroprotective effects indirectly by regulating gut microbiota. The beneficial health effects of polyphenols mainly depend on their bioavailability and absorption rate [130]. A large proportion of phenolic compounds are not directly absorbed and remain in the colon to be metabolized by gut microbiota, where they are converted into polyphenolic metabolites with higher bioavailability by gut microorganisms [131]. Interestingly, when administered in combination with oat β-glucan, polyphenols were shown to regulate the gut microbiota community phenotype and increased probiotics in high-fat-diet fed mice [132]. Accordingly, it was recently reported that polyphenol enriched oat extracts increased the proliferation of beneficial gut microbiota, showing a positive correlation between antioxidant activity and prebiotic effect, which suggest that these polyphenols could be used to regulate the gut microbiota composition [131]. In this sense, certain changes in gut microbiota have been described to increase the risk of a cerebrovascular event, and, inversely, stroke can induce dysbiosis [133]. Therefore, a therapeutic effect of phenolic acids and flavonoids by the modulation of gut microbiota in experimental models is plausible and future studies targeting polyphenols–gut interplay are warranted.

It is well known that consumption of certain foods, such as cereals, fruit or vegetables, can reduce many risk factors related to neurovascular disease [134]. The wide range of health benefits of polyphenols has drawn the attention of the food industry and many phenolic acids and flavonoids found in *S. ramosissima* are also available commercially as dietary supplements with different health claims. 

Phenolic acids are easily absorbed by the gastrointestinal tract [135,136] and some of the aforementioned compounds are marketed as food products. As an example, cinnamic acid is marketed in liquid formulation as oral drops intended to support bladder dysfunction, cystitis or asthma, among other conditions, and ferulic acid tablets are recommended as antioxidants. In addition, sitostanol is used as an ingredient in food products designed to lower cholesterol levels and both the Food and Drug Administration (FDA) and the European Food Safety Authority (EFSA) allows manufacturers of products that contain sito-stanol to claim this health benefit.

Most compounds within the flavonoid group are marketed as herbal extracts or natural isolated compounds from different plants. Phloretin is marketed as a nutricosmetic, a dietary supplement capsule containing green apple extract to support skin health. Several flavanols supplements from green tea extract (catechin, epicatechin, epigallocatechin) are intended for boost sport performance. In addition, Taxifolin tablets are commercialized as a help to improve venous circulation. Narigining and apigenin are suggested, among other uses, for improving cognitive function. Chrysin has been shown to be an inhibitor of aromatase enzyme activity [137] and is used as a phytoestrogen to regulate the conversion of testosterone into estrogen. Naturally extracted luteolin, quercetin and rutin are used to fight oxidative stress and inflammation and restore immune health. However, it is well known that the low bioavailability of dietary flavonoids is a major limitation for their use [138]. 

In addition, some polyphenolic-rich mixtures are also available as botanical extracts, such as tea, coffee or berry extracts.

**Figure 1:**
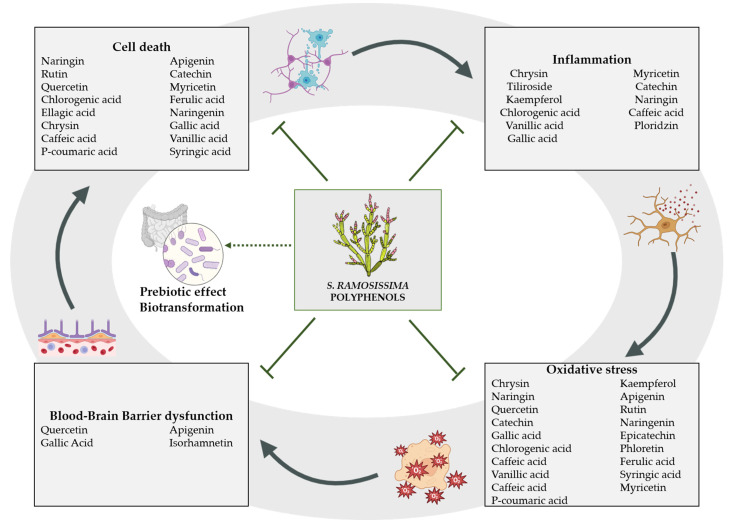
Ischemic stroke pathological mechanisms modulated by phenolic acids and flavonoids identified in *S. ramosissima.* As free radical scavengers, polyphenols counteract ROS generation and the inflammation triggered by oxidative stress. Some phenolic acids and flavonoids can directly act on endothelial cells and reduce BBB dysfunction or modulate mechanisms leading to cell death that prevent neuronal loss. Additionally, phenolic acids and flavonoids may have indirect beneficial effects by favoring the increase of beneficial bacteria, which in turn modulate their metabolisms and bioavailability.

Despite clinical trials showing that polyphenol supplements are safe to consume and well tolerated [139], to our knowledge there is only one human study assessing a *S. ramosissima* extract. Authors reported an inhibitory effect of Salicornia on hyperkinesis, an analgesic effect and a role on skin barrier architecture [139,140,141]. However, this study investigated *S. ramosissima* as a skin cream application and further clinical studies are needed on the oral safety and tolerability of *S. ramosissima* polyphenol-rich extracts in humans. In this sense, preclinical studies support both the safety [142] and the use of *S. ramosissima* polyphenolic profile for the treatment of ischemia [13,143]. Moreover, Salicornia extracts from other Salicornia species have been better studied in neuroprotection, such as *S. europaea* [144] or *S. herbacea* [145]. Altogether, since neurodegenerative processes develop not only in the acute stage of ischemia, but also progress throughout the survival period after ischemia [146], *S. ramosissima* polyphenolic profile may be a promising complementary agent in the future against the development of post-ischemic brain neurodegeneration, as depicted in Figure 1.

## Figures and Tables

**Table 1:** Polyphenolic compounds identified in *S. ramosissima*.

Polyphenol	Subclass	Compound	Ref.
Flavonoid	Dihydrochalcone	Phloretin	[20]
		Phloridzin	[20]
	Flavanol	Catechin	[20]
		Epicatechin	[20]
		(Epi)gallocatechin	[13]
		Dihydroquercetin (Taxifolin)	[21]
	Flavanone	Naringin	[20]
		Naringenin	[20]
	Flavone	Apigenin	[20]
		Apigenin-6-arabinosyl-8-glucoside (isoschaftoside)	[21]
		Chrysin	[20]
		Luteolin glucosyllactate	[13]
	Flavonol	Isorhamnetin	[22]
		Isorhamnetin 3-glucoside	[22]
		Isorhamnetin-7-O-(6-O-malonyl)-glucoside	[23]
		Isorhamnetin glucopyranoside	[13]
		Kaempferol	[20]
		kaempferol derivative	[21]
		kaempferol-3-O-glucoside	[20]
		kaempferol-3-O-rutinoside	[20]
		Myricetin	[20]
		Quercetin	[20]
		Quercetin-3-O-galactoside	[20]
		Quercetin glucoside	[13]
		Quercetin 3-glucoside (Isoquercitrin)	[21,22,23]
		Quercetin-malonyglucoside	[13,21]
		Quercetin-methyl-ether derivative (isomer 1 and 2)	[21]
		Quercetin-rhamnosyl-hexoside	[13,21]
		Rutin (quercetin 3 -O rhamnosyl glucoside, quercetin rutinoside, vitamin p)	[20]
Phenolic acids	Hydroxybenzoic acids	Cannabidiolic acid	[13]
		Salicylic acid derivative	[21]
		Sitostanol	[24]
		Syringic acid	[20]
		Tiliroside	[20]
		Vanillic acid	[20]
		Ellagic acid	[20]
		Gallic acid	[20]
		Gallocatechin	[24]
		Protocatechuic acid	[20]
		Protocatechuic-arabinoside acid	[21]
	Hydroxycinnamic acids	Cinnamic acid	[25]
		*P*-coumaric acid (4-hydroxycinnamic acid)	[13,20,21,23]
		Sinapic acid (3,5-Dimethoxy-4-hydroxycinnamic acid)	[20]
		Ethyl (E)-2-hydroxycinnamate	[24]
		*P*-coumaric acid benzyl ester derivative	[21]
		Quinic acid	[13,21,23]
		*P*-coumaroylquinic acid (isomer 1 and 2)	[21]
		Caffeic acid	[20,22]
		Hydrocaffeic acid	[22]
		Caffeic acid-glucuronide-glucoside (isomer 1)	[21]
		Caffeoylquinic acid	[22]
		Chlorogenic acid (3-O-caffeoylquinic acid)	[20,21,23]
		Neochlorogenic acid (5-O-caffeoylquinic acid)	[13,21]
		Dicaffeoylquinic acid (isomer 1, 2, 3 and 4)	[13,22]
		3,4-Di-O-caffeoylquinic acid	[20,22]
		3,5-Di-O-caffeoylquinic acid	[20]
		3,5-Dicaffeoylquinic acid	[21]
		4,5-Dicaffeoylquinic acid	[21]
		Hydrocaffeoylquinic acid	[13,21,22]
		Dihydrocaffeoyl quinic acid	[22]
		Caffeoyl-hydrocaffeoyl quinic acid	[21,22]
		Tungtungmadic acid (3-Caffeoyl-4-dihydrocaffeoyl quinic acid) (isomer 1 and 2)	[13]
		Ferulic acid	[13,21,23,25]
		Ferulic-glucoside acid	[21]
		Trans-ferulic acid	[20]
	Coumarin	Scopoletin	[13,24]

## Data Availability

No applicable.
